# Paracentral acute middle maculopathy following coronary angiography^☆^

**DOI:** 10.1016/j.ajoc.2022.101674

**Published:** 2022-08-13

**Authors:** Kenneth J. Schmitt, Rajat Agrawal, Sean D. Adrean

**Affiliations:** a University of California, Irvine School of Medicine, Irvine, CA, USA; b Retina Global, Aliso Viejo, CA, USA; c Retina Consultants of Orange County, Fullerton, CA, USA

**Keywords:** Paracentral acute middle maculopathy, PAMM, Coronary angiography

## Abstract

**Purpose:**

To describe a case of paracentral acute middle maculopathy (PAMM) after coronary angiography*.*

**Observations:**

A 65-year-old female patient exhibited a dense central scotoma 5 hrs after coronary angiography. She presented the next day to the retina clinic and received a complete visual examination including slit lamp biomicroscopy, dilated fundus examination, fluorescein angiogram (FA), spectral domain optical coherence tomography (SD-OCT) and OCT-Angiography (OCT-A). She was found to have the characteristic findings of PAMM including a hyperreflective band at the inner nuclear layer (INL) with extension into the inner plexiform layer (IPL) and outer plexiform layer (OPL) on imaging.

**Conclusions and Importance:**

PAMM lesions can occur immediately following coronary angiography. The acute nature of the presentation and time to examination in this case lend further insights into the pathophysiology of PAMM. When patients undergo cardiovascular interventions and report new onset visual scotomas, the diagnosis of PAMM should be considered with referral for careful ophthalmic examination and work-up.

## 1 Introduction

Paracentral acute middle maculopathy (PAMM) was initially recognized as a superficial variation of acute macular neuroretinopathy (AMN), characterized by paracentral scotomas associated with middle retinal involvement either above (type 1) or below (type 2) the OPL.^1^ Type 2 lesions are consistent with previous descriptions of AMN. However, more recent literature has recognized PAMM, the type 1 lesions, to be a distinctly independent condition from AMN, citing differences in frequency of occurrence, demographic disparities, and various risk factors.^2^ PAMM has been more recently defined as a hyperreflective, thickened band involving the inner nuclear layer (INL) with or without extension into the inner and outer plexiform layers (IPL, OPL).^1^^,^^3^ Such lesions indicate an ischemic insult of the deep vascular complexes (DVC) and with the resolution, typically have a characteristic thinning of the INL.

Since its original description in 2013, the understanding of PAMM continues to evolve. Although some studies have alluded to PAMM presenting more frequently amongst the elderly, others have considered it to be idiopathic and likely to present amongst young, healthy individuals.^2^ Nonetheless, the detection of PAMM should prompt a comprehensive examination of possible contributing systemic or extrinsic risk factors, both of which continue to be explored and studied in regard to PAMM.

The purpose of this paper is to report a unique case of a patient who presented with a paracentral scotoma immediately after coronary angiography and was confirmed to be a case of PAMM based on the pathognomonic OCT findings.

## 2 Case report

A 65-year-old woman with a medical history of mitral valve prolapse and regurgitation presented to her cardiologist with atypical chest pain, palpitations and shortness of breath, prompting a diagnostic left and right heart catheterization via the right common femoral artery and vein respectively. No stents were required, and she had normal coronary morphology. She tolerated the procedure well without complications. At home, 5 hrs after the procedure, she noticed an acute paracentral scotoma in the left eye. She initially observed this loss of vision only when she closed her right eye. She described it as a purplish box shape spot superonasal to fixation. The patient was seen at the retina clinic the next morning, 18 hours after the symptoms were first noticed. She denied any recent flu-like illnesses or hypotensive episodes. She reported occasional alcohol use, no tobacco use, and reported minimal caffeine consumption (1–3 times/week). She also denied taking any pressor or sympathomimetic medication at the time of evaluation.

The patient's ocular history was significant for bilateral retinal tears that were previously treated.

Upon presentation, the patient's best-corrected visual acuity was 20/20 in the right eye and 20/25 in the left eye. No relative afferent papillary defect was present. Intraocular pressure was 12 mmHg in the right eye and 13 mmHg in the left eye. The patient had mild nuclear sclerotic cataracts OU.

**Fig. 1:**
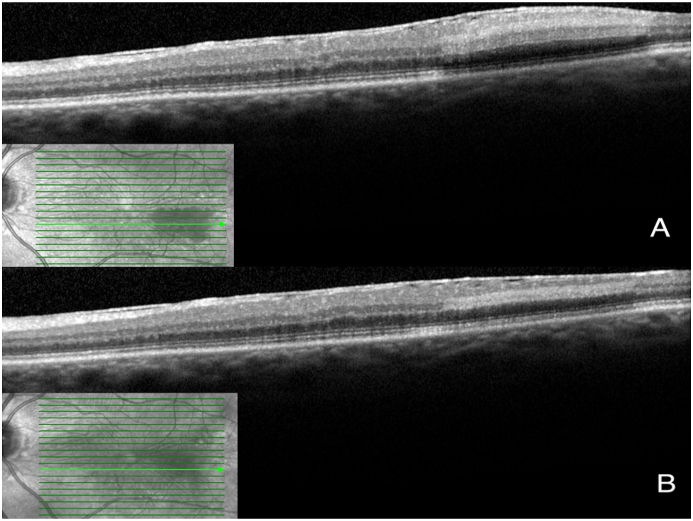
(A) Near-infrared reflectance (NIR) with corresponding spectral domain optical coherence tomography (SD-OCT) of the left eye on initial presentation. Note the hyperreflective band at the inner nuclear layer (INL) with extension into the inner plexiform layer (IPL) and outer plexiform layer (OPL). A mild ERM is present. (B) Near-infrared reflectance (NIR) with corresponding spectral domain optical coherence tomography (SD-OCT) of the left eye at two-week follow up.

**Fig. 2:**
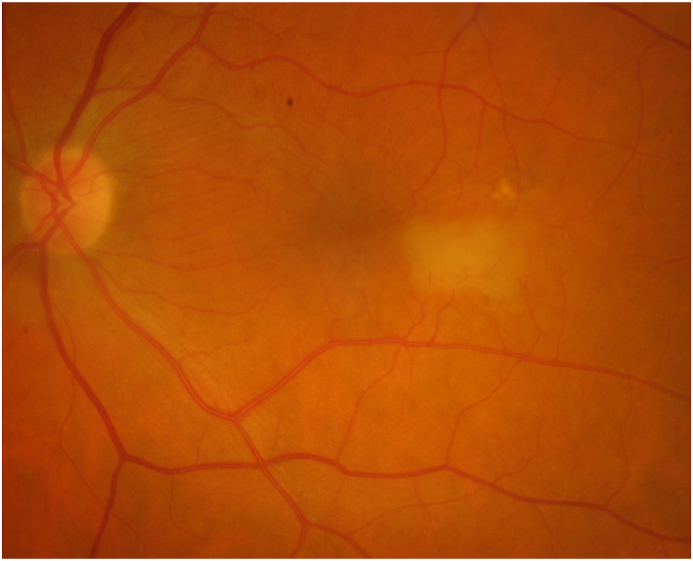
Color fundus photography of the left eye upon initial visit illustrates apparent white discoloration located nasally to the fovea. (For interpretation of the references to color in this figure legend, the reader is referred to the Web version of this article.)

**Fig. 3:**
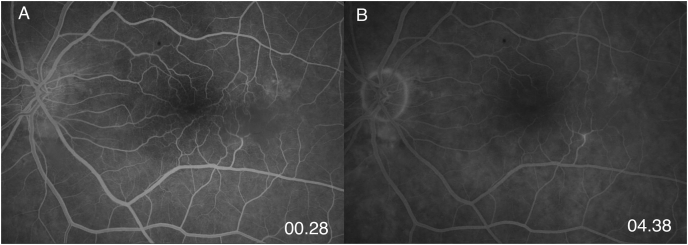
Fluorescein angiography (FA) of the left eye shows a blockage of a twig retinal artery with late vascular staining: (A) 0.28 min (B) 4.38 min.

**Fig. 4:**
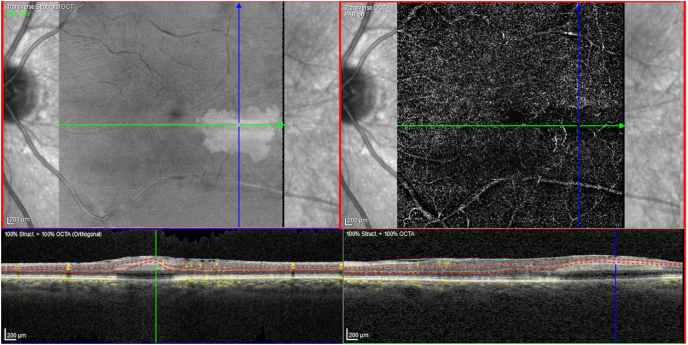
OCT Angiography of the left eye retinal vessels shows significant capillary dropout in the deep retinal capillary plexus (DCP).

**Fig. 5:**
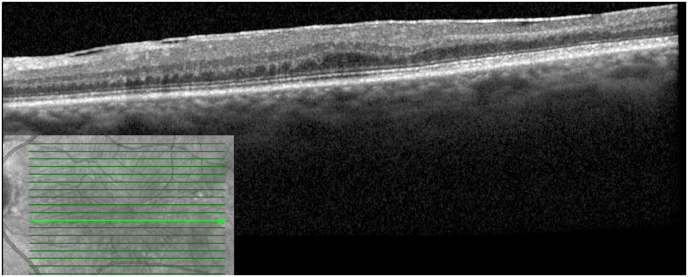
Near-infrared reflectance (NIR) with corresponding spectral domain optical coherence tomography (SD-OCT) of the left eye at three months follow up. Note reduced hyperreflectivity at the inner nuclear layer (INL) with continued INL thinning.

On fundoscopy, the right eye had a posterior vitreous detachment (PVD) with a cup to disc ratio of 0.1. There was a mild epiretinal membrane overlying the fovea. The vessels were normal. Superotemporally, there were two retinal holes that were well surrounded with laser. The left eye had a PVD, with a cup to disc ratio of 0.1. There was a mild epiretinal membrane overlying the fovea. Temporal and slightly inferior to the fovea, there was a 2 disc by 1 disc area of retinal whitening. Vessels were normal, with no emboli seen. There was a chorioretinal scar present superotemporally from previous cryotherapy. SD-OCT showed a central thickness of 322 μm in the right eye and 383 μm in the left eye with typical middle retinal ischemia (Fig. 1). Fundus photos showed retinal whitening in the area of the PAMM lesion (Fig. 2). Fluorescein angiogram (FA) revealed early blockage with delayed filling, with late staining of the retinal arteriole (Fig. 3a–b). OCT-A exhibited deep capillary non-perfusion (Fig. 4). At the three-months follow-up, the patient reported minimal improvement of visual symptoms but with a continual area of a visual scotoma and a corresponding purple spot in the left eye (Fig. 5). The best corrected visual acuity was 20/20 in each eye.

## 3 Discussion

The occurrence of PAMM has been associated with various vasoconstricting agents including sympathomimetics agents such as epinephrine, norepinephrine, and caffeine.^4^ Additionally, PAMM has been seen with a number of vascular diseases including diabetic retinopathy, sickle cell retinopathy, Purtscher's retinopathy, migraines, retinal artery and retinal vein occlusions.^4^^,^^5^ In this case report, PAMM was seen after a coronary angiogram.

The vasculature of the macula has been well documented with the superficial (SCP), intermediate (ICP) and deep (DCP) retinal capillary plexuses described to be organized in a vertical or in series arrangement, with the majority of afferent arterial flow and efferent venous outflow occurring at the SCP and DCP respectively.^3^ Of note, the DCP supplies the middle retina and maintains a lower perfusion pressure since it is perfused later in the serial structure of retinal capillary plexuses. The DCP has also been shown to have some of the highest oxygen demand due to increased metabolic activity of the ONL.^3^ Given this, the DCP has previously been proposed to reside in a watershed zone with the combination of decreased perfusion pressure and increased oxygen demand creating a condition that can result in a selective ischemic insult in the middle retina or INL with possible extension to the IPL and OPL.^6^ Collectively, this supports the notion that the DCP within the INL is at one of the highest risks for ischemic insult, with increasing severity of occlusion noted by additional inner retinal layer involvement.

The etiology of PAMM is thought to be partially explained by this ischemic cascade and represents one of the mildest forms within this proposed spectrum of ischemia. PAMM has regularly been reported to align with the intermediate (ICP) and deep capillary plexuses (DCP), supporting the hypothesis of PAMM being secondary to the aforementioned deep capillary ischemia.^3^ The characteristic development of INL thinning following regression of the original PAMM lesion further denotes the primary etiology of being a milder ischemic insult given the selective retinal layer perfusion.

The present case describes a patient found to have PAMM in the left eye shortly after undergoing a coronary angiogram. PAMM has characteristic OCT findings associated with multiple systemic and external risk factors. There have been reported associations with cardiovascular interventions and complications including angioplasty with coronary stent placement and cardiac arrest, despite stent placement being shown to associate with AMN, the type 2 lesion, rather than a characteristic PAMM lesion.^7^^,^^8^ To the best of our knowledge, there has been no report of PAMM following coronary angiography.

Several factors could have possibly contributed to the patient's development of PAMM. First, although of low incidence, coronary angiography with left heart catherization has rarely been linked to cerebrovascular complications: cerebral micro embolisms are thought to be the primary mechanism.^9^ Consequently, vascular trauma from heart catherization, especially in the aorta, may cause the release of micro embolisms into systemic circulation and subsequent partial occlusion of the retinal capillary vasculature, leading to the characteristic anatomic findings of PAMM. Additionally, while there have been several case reports associating mitral valve prolapse (MVP) to retinal and cerebral emboli, larger comparative studies have found no association with MVP to cerebral ischemic attacks.^10^, ^11^, ^12^ It is also likely that more invasive cardiac procedures would increase the chances of micro embolism of retinal capillary vasculature and subsequent visual sequelae. Fluorescein angiogram in our patient showed a probable reperfused twig retinal artery occlusion in an inferior retinal arteriole, noted by delayed filling and late staining of the vessel on FA aligning with the area of the ischemic insult.

The PAMM lesion for our patient was also noted to occur in the area between the superior and inferior retinal vascular arcade, along the horizonal raphe (HR), which has also been described as a watershed zone.^13^ Given the etiology of PAMM, our patient may represent a unique demonstration of a dual watershed effect due to both the vertical intraretinal location of the affected DCP, and the horizontal positioning within the HR.^6^^,^^13^

The patient's paracentral loss of vision persisted through the three-months follow-up, which commonly occurs in PAMM, with permanent vision scotomas occurring in most patients.^2^

## 4 Conclusion

PAMM can occur immediately following coronary angiography. When patients undergo cardiovascular intervention and subsequently complain of visual paracentral scotomas, the diagnosis of PAMM should be considered with referral for careful ophthalmic examination and work-up.

## Funding

No funding or grant support.

## Patient consent

The patient orally consented to the publication of this case. This report does not contain any personal information that could lead to the identification of the patient.

## Authorship

All authors attest that they meet the current ICMJE criteria for Authorship.

## Declaration of competing interest

None.
